# Lenalidomide overcomes suppression of human natural killer cell anti-tumor functions by neuroblastoma microenvironment-associated IL-6 and TGFβ1

**DOI:** 10.1007/s00262-013-1466-y

**Published:** 2013-08-27

**Authors:** Yibing Xu, Jianping Sun, Michael A. Sheard, Hung C. Tran, Zesheng Wan, Wei Yao Liu, Shahab Asgharzadeh, Richard Sposto, Hong Wei Wu, Robert C. Seeger

**Affiliations:** 1 Division of Hematology/Oncology, Children’s Hospital Los Angeles, 4650 Sunset Boulevard, Mailstop #57, Los Angeles, CA 90027 USA; 2 Keck School of Medicine, University of Southern California, Los Angeles, CA USA

**Keywords:** Neuroblastoma, NK cells, ADCC, Lenalidomide, IL-6, TGFβ1

## Abstract

**Background:**

Treatment for children with high-risk neuroblastoma with anti-disialoganglioside mAb ch14.18, IL-2, and GM-CSF plus 13-*cis*-retinoic acid after myeloablative chemotherapy improves survival, but 40 % of patients still relapse during or after this therapy. The microenvironment of high-risk neuroblastoma tumors includes macrophages, IL-6, and TGFβ1. We hypothesized that this microenvironment suppresses anti-tumor functions of natural killer (NK) cells and that lenalidomide, an immune-modulating drug, could overcome suppression.

**Methods:**

Purified NK cells were cultured with IL-2, neuroblastoma/monocyte-conditioned culture medium (CM), IL-6, TGFβ1, and lenalidomide in various combinations and then characterized using cytotoxicity (direct and antibody-dependent cell-mediated cytotoxicity), cytokine, flow cytometry, and Western blotting assays. Anti-tumor activity of NK cells with lenalidomide, ch14.18, or both was evaluated with a xenograft model of neuroblastoma.

**Results:**

CM from neuroblastoma/monocyte co-cultures contains IL-6 and TGFβ1 that suppress IL-2 activation of NK cell cytotoxicity and IFNγ secretion. IL-6 and TGFβ1 activate the STAT3 and SMAD2/3 pathways in NK cells and suppress IL-2 induction of cytotoxicity, granzymes A and B release, perforin expression, and IFNγ secretion. Lenalidomide blocks IL-6 and TGFβ1 activation of these signaling pathways and inhibits their suppression of NK cells. Neuroblastoma cells in NOD/SCID mice exhibit activated STAT3 and SMAD2/3 pathways. Their growth is most effectively inhibited by co-injected peripheral blood mononuclear cells (PBMC) containing NK cells when mice are treated with both ch14.18 and lenalidomide.

**Conclusion:**

Immunotherapy with anti-tumor cell antibodies may be improved by lenalidomide, which enhances activation of NK cells and inhibits their suppression by IL-6 and TGFβ1.

**Electronic supplementary material:**

The online version of this article (doi:10.1007/s00262-013-1466-y) contains supplementary material, which is available to authorized users.

## Introduction

Addition of anti-disialoganglioside (GD2) chimeric mAb ch14.18, IL-2, and GM-CSF immunotherapy to 13-*cis*-retinoic acid improved event-free survival (EFS) for patients with high-risk neuroblastoma, but approximately 40 % still relapsed during or after this therapy [1]. An immunosuppressive tumor microenvironment could negatively impact the anti-tumor cell activity of antibody therapy that is largely reliant upon antibody-dependent cellular cytotoxicity (ADCC) mediated by natural killer (NK) cells. Our gene expression analyses of high-risk, metastatic neuroblastomas without MYCN gene amplification using Affymetrix^®^ microarrays and TaqMan^®^ low-density arrays revealed that high expression of genes related to inflammation, including IL-6, IL-6R, and TGFβ1, was associated with a poor 5-year EFS [2–4]. Furthermore, we found that high-risk primary neuroblastoma tumors contain CD68+ tumor-associated monocytes/macrophages (TAMs) producing IL-6 and that bone marrows with neuroblastoma metastases include CD33+ CD14+ monocytic cells, which also express IL-6 [3]. Experimentally, we have shown that neuroblastoma cells stimulate blood monocytes to secrete IL-6 and that TAMs stimulate the growth of neuroblastoma cells in NOD/SCID mice, at least partially via IL-6 secretion [3].

IL-6, which induces activation of the STAT3 signaling pathway, has not been definitively shown to affect the function of human NK cells. Treatment for patients with recombinant human IL-6 has been reported to suppress the ability of their peripheral blood mononuclear cells (PBMC) to kill K562 cells in vitro [5]. Although K562 cells are sensitive to NK cell cytotoxicity, this study did not demonstrate a direct effect of IL-6 upon NK cells or any other possible mechanism [5]. The potential importance of STAT3 pathway activation in suppressing NK cell cytotoxicity is supported by experiments showing that NK cells from tumor-bearing mice with induced ablation of STAT3 signaling are more cytotoxic against T cell lymphoma YAC-1 cells, which are NK cell-sensitive, than those from tumor-bearing mice with wild-type STAT3 [6].

TGFβ1 activates the SMAD2/3 signaling pathway in human NK cells, which results in the suppression of ADCC and secretion of IFNγ, TNFα, and GM-CSF in vitro [7–9]. Culture of blood NK cells with TGFβ1 decreases the expression of CD16 after 24 h [10, 11]. NK cells from patients with glioblastoma, lung, and colorectal cancer have decreased the expression of the activating immunoreceptor NKG2D, and TGFβ1 in serum and cerebrospinal fluid is responsible for this down-regulation [12, 13].

Based upon the above observations, we hypothesized that IL-6 and TGFβ1 in the neuroblastoma microenvironment may contribute to the failure of treatment with mAb ch14.18 by suppressing anti-tumor functions of NK cells. Using purified NK cells, we found that anti-tumor NK cell functions induced in vitro by IL-2, including direct cytotoxicity, ADCC, and IFNγ secretion, are suppressed by conditioned medium (CM) from neuroblastoma/monocyte co-cultures that contain IL-6 and TGFβ1 and that suppression is removed by the depletion of these cytokines from the CM. Additionally, these recombinant cytokines suppressed IL-2 activation of NK cells.

Lenalidomide is an immune-modulating drug that activates T cells to secrete IL-2, which in turn activates NK cell cytotoxicity and ADCC [14, 15]. Clinical trials in children and adults demonstrated a greater number of NK cells and increased cytotoxicity, decreased T regulatory cells, and increased secretion of IL-2, IL-15, and GM-CSF after 21 days of lenalidomide treatment [16, 17]. Preclinical in vitro data have shown that lenalidomide can enhance ADCC mediated by human NK cells with rituximab (anti-CD20) against lymphoma and chronic lymphocytic leukemia cells [18–20], with SGN-40 (anti-CD40) against multiple myeloma and chronic lymphocytic leukemia cells [21, 22], and with trastuzumab (anti-HER2/neu) and cetuximab (anti-EGFR) against solid tumor cell lines [23]. Because of these clinical and preclinical effects, we hypothesized that lenalidomide also could prevent the suppression of IL-2 activation of NK cells by neuroblastoma/monocyte CM, IL-6, and TGFβ1. Indeed, we show that lenalidomide blocks the activation of STAT3 and SMAD2/3 signaling pathways and prevents the suppression of NK cell activation in vitro. Further, NK cells among PBMC that are co-injected with neuroblastoma cells into NOD/SCID mice have the greatest anti-tumor effect when mice are treated with both lenalidomide and mAb ch14.18.

## Materials and methods

For additional details on “Materials and methods,” see Supplemental Methods.

### Reagents

Details for reagents are provided in Supplemental Methods. Anti-GD2 chimeric mAb ch14.18 was provided by the National Cancer Institute-Frederick. Lenalidomide was provided by Celgene and was dissolved in dimethyl sulfoxide (final DMSO concentration for in vitro and in vivo experiments was 0.03 and 5 %, respectively).

### Neuroblastoma cell lines and tumors

The CHLA-255 parent cell line, the firefly luciferase (Fluc) and humanized Renilla luciferase (hRluc) transfected CHLA-255 cell lines, and the LA–N-1 cell line were cultured in IMDM with 15 % FBS. Primary untreated tumors from patients with high-risk, stage 4 (metastatic) neuroblastoma were obtained from patients enrolled and consented in therapeutic and biology protocols of the Children’s Oncology Group (COG).

### NK cells, monocytes, and cell culture

PBMC were isolated from blood of normal adult donors by density separation using Ficoll-Hypaque [24]. NK cells and monocytes were isolated from PBMC by negative selection using a NK Cell Isolation Kit or a Monocyte Cell Isolation Kit, an LS Column, and a MidiMACS™ Separator according to the manufacturer’s protocol (Miltenyi Biotec). Purified NK cells (CD56+ CD16+ CD3−) were >95 %, and purified monocytes (CD14+) were >95 %. NK cells were removed from PBMC by positive selection using CD56 MicroBeads and the column and separator mentioned above (CD56+ was <0.02 %; Fig. 6 legend). Purified NK cells (1 × 10^4^) were cultured in 96-well black Costar plates (Corning, Inc.) in 100 μl RPMI-1640 (Invitrogen) with 15 % FBS in the presence of IL-2 (10 ng/ml). CM for testing with NK cells was generated with 72-h cultures of monocytes (10^6^/ml), CHLA-255-Fluc neuroblastoma cells (10^6^/ml), or CHLA-255-Fluc/monocytes together (10^6^/ml of each cell type) in IMDM and 15 % FBS. Blood from normal adult donors was obtained in accordance with a protocol approved by the Committee on Clinical Investigation (CCI) at Children’s Hospital Los Angeles.

### Cytotoxicity assay

For cytotoxicity assays, two neuroblastoma cell lines (LA–N-1 and CHLA-255-Fluc) were labeled with calcein-AM for 30 min [25] and then 5 × 10^3^ tumor cells were added to NK cells that had been cultured for 72 h at an effector-to-target (E:T) cell ratio of 2:1 with or without mAb ch14.18 (0.1 μg/ml). These cells were then co-incubated for 6 h at 37 °C, and surviving tumor cells were quantified as calcein-containing cells with a digital imaging microscopy system (DIMSCAN), a fluorescence-based multi-well assay, as previously described [25].

### TaqMan^®^ low-density array (TLDA) assays, flow cytometry, cytometric bead array (CBA), Luminex^®^, ELISA assays, and Western blotting

Details are provided in Supplemental Methods.

### Murine model of neuroblastoma

Female NOD/SCID mice that were 4 weeks old were purchased from The Jackson Laboratory. Rat anti-mouse CD122/IL-2Rβ (200 mg/mouse) was injected intraperitoneally 1 day before tumor cell injection to eliminate residual murine NK cells [26]. CHLA-255-Fluc neuroblastoma cells and human PBMC were co-injected subcutaneously near both shoulders in 25 % (v/v) BD Matrigel™ Matrix Growth Factor Reduced (BD Biosciences). Tumor growth was assessed by bioluminescence imaging using a Xenogen IVIS^®^ 100 instrument. Animal experiments were done in accordance with a protocol approved by the Institutional Animal Care and Usage Committee of Children’s Hospital Los Angeles.

### Statistical analysis

Data were analyzed using Stata statistical software (version 11.2) and are represented as mean ± SD unless otherwise stated. Two-tailed Student’s independent sample *t* test was performed to determine the significance of the observed differences. A *P* value of <0.05 was considered significant. Analysis of tumor bioluminescence data transformed the average photon flux of two tumors for each mouse using the log (flux + 1) transformation and then calculated the area under the growth curve (AUC). The AUC values were used in the analysis to compare differences in tumor photon flux between treatment groups. Mouse survival time is defined as the length of time (in days) from the tumor cell injection date until the mouse is killed due to tumor size (1.5 cm diameter) or end of the study. Linear regression was utilized to determine any differences in the AUC due to cell type (PBMC versus PBMC-depleted NK cells) and treatment groups (lenalidomide, ch14.18, or lenalidomide with ch14.18). Similarly, survival time was evaluated using censored normal regression.

## Results

### High level expression of mononuclear phagocyte-associated genes, IL-6, IL-6R, IL-10, and TGFβ1 in neuroblastoma tumors from patients

**Fig. 1:**
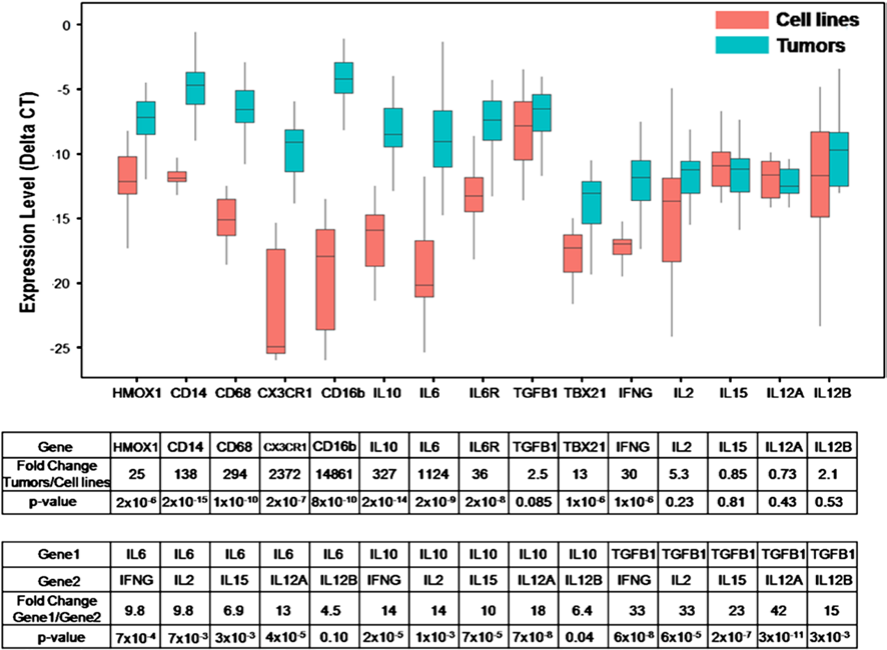
Expression of monocyte/macrophage and cytokine genes in neuroblastoma tumors and cell lines. Expression of genes in primary untreated high-risk, metastatic neuroblastomas (*n* = 38) and in neuroblastoma cell lines (*n* = 23) was quantified with a TaqMan^®^ low-density array assay. Normalized expression (ΔCT) of monocyte/macrophage-associated genes (HMOX1, CD14, CD68, CX3CR1, and CD16b), of IL-10, IL-6, IL-6R, TGFβ1, and TBX21, and of NK cell growth, survival, and activation genes (IL-2, IL-15, IL-12A, IL-12B, and IFNγ) are shown. Fold change in gene expression by tumors as compared to cell lines was calculated using 2^(ΔCT of Tumor − ΔCT of Cell lines)^. A comparison of expression of IL-6, IL-10, and TGFβ1 versus IFNγ, IL-2, IL-15, IL-12A, and IL-12B in neuroblastoma tumors is shown in the lower table

Gene expression of MYCN-amplified and non-amplified stage 4 (metastatic) neuroblastoma tumors and cell lines was assessed using TLDA assays. Monocyte-associated genes such as CD14, CD16, CD68, and HMOX1 as well as IL-6, IL-6R, and IL-10 were expressed by tumors, whereas their expression was significantly lower in cell lines (Fig. 1). In contrast, both tumors and cell lines expressed TGFβ1 at high and near-equal levels, and genes that are directly and indirectly regulated by TGFβ1 (i.e., TBX21/TBET and IFNγ) were weakly expressed in tumors (Fig. 1). IL-2, IL-15, IL-12A/p35, and IL-12B/p40, which are important for NK cell proliferation, differentiation, and activation [27], were weakly expressed by tumors and cell lines. Comparing the expression of IL-6, IL-10, and TGFβ1 to that of IL-2, IL-15, IL-12A, IL-12B, and IFNγ demonstrated 5- to 42-fold greater levels of the former than the latter (Fig. 1). These data suggest that neuroblastoma tumors are rich in potentially immunosuppressive cytokines (IL-6 and TGFβ1), but not in cytokines that support NK cell proliferation, differentiation, and activation (IL-2, IL-15, IL-12A, and IL-12B).

### Neuroblastoma cell line cultures release TGFβ1 and neuroblastoma/monocyte co-cultures release TGFβ1, IL-6, and IL-10

**Fig. 2:**
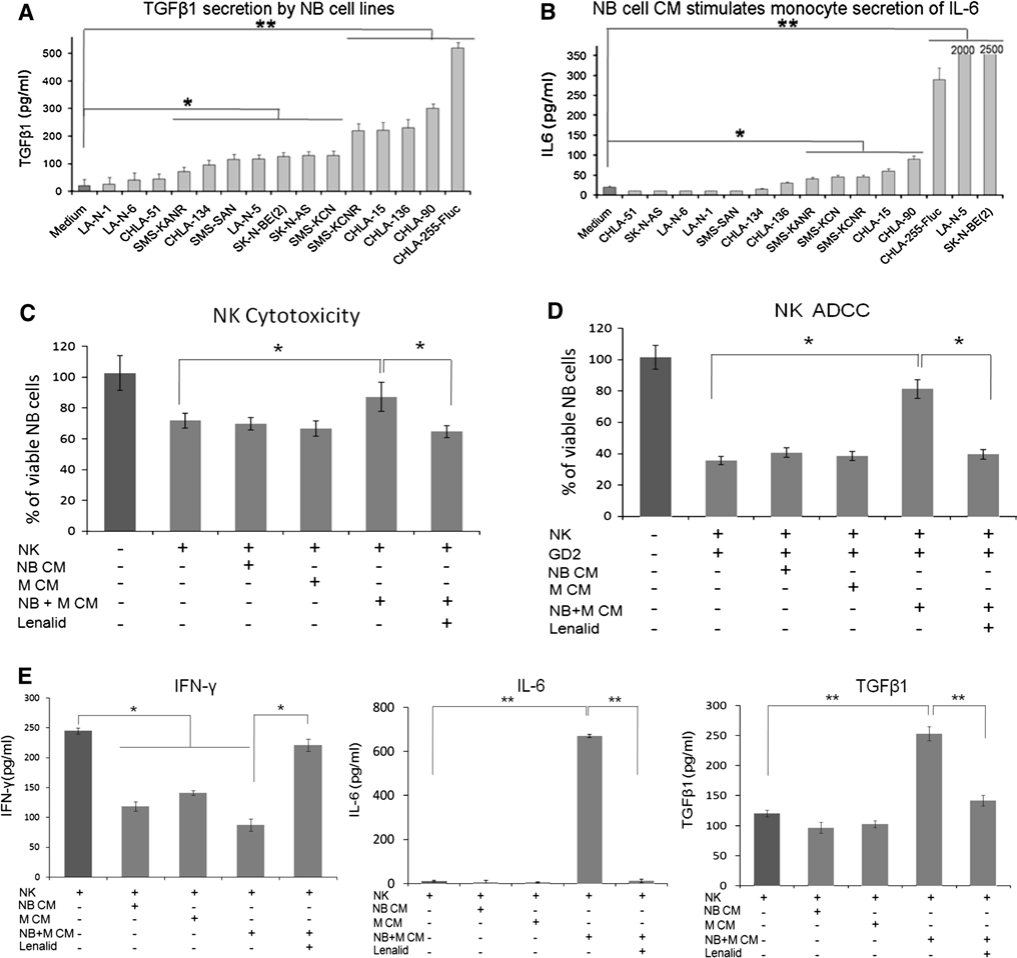
Suppression of IL-2 induction of NK cell direct cytotoxicity, ADCC, and IFNγ secretion by neuroblastoma/monocyte-conditioned medium is prevented by lenalidomide. **a** Fifteen neuroblastoma cell lines (5 × 10^5^ cells/ml) were cultured for 72 h, and then, the total TGFβ1 was quantified in the CM by ELISA (mean ± SD for 3 replicate cultures). **b** Purified monocytes (5 × 10^5^ cells/ml) were cultured for 24 h with CM generated after 72 h by fifteen neuroblastoma cell lines (5 × 10^5^ cells/ml), and then IL-6 was quantified in monocyte supernatants by ELISA (mean ± SD for 3 replicate cultures). Cell lines alone did not secrete IL-6. The *t* test *P* values for medium alone versus CM *P < 0.05; **P < 0.01. **c**, **d** NK cells isolated from PBMC were cultured (1 × 10^4^ cells/0.1 ml/well) for 72 h with IL-2 alone (10 ng/ml), with added CM (50 % v/v) from monocytes (*M*), CHLA-255-Fluc neuroblastoma cells (NB), or neuroblastoma/monocyte co-culture (N + M CM), and with lenalidomide (10 μM) as indicated, and then direct cytotoxicity and ADCC (*E*:*T* ratio = 2:1) with ch14.18 (0.1 μg/ml) were quantified after 6 h of co-culture with CHLA-255-Fluc neuroblastoma cells using the calcein-AM/DIMSCAN assay (mean ± SD for 8 replicate cultures for each condition). The M, NB, and M + NB CMs had 312, 159, and 412 pg/ml TGFβ1; 11, 37, and 556 pg/ml IL-6; and 12, 14, and 13 pg/ml of IFNγ, respectively. **e** IFNγ, IL-6, and TGFβ1 were quantified by ELISA in the culture media from these same NK cell cultures at 72 h, and the amount of each cytokine contributed by NK cells was calculated (NK cytokine = total cytokine − CM cytokine ÷ 2). Confirmatory results were obtained from 1 additional experiment. The *t* test *P* values *P < 0.05; **P < 0.01

TGFβ1 was released into the culture medium by 12 of 15 well-characterized neuroblastoma cell lines [28–31], as assessed by ELISA analysis of CM after 72 h (Fig. 2a). Culture of purified monocytes with 72-h tumor cell line CM showed that 6 of 15 CM also stimulated the secretion of TGFβ1 by monocytes after 24 h (data not shown). In contrast, IL-6 was not released by neuroblastoma cell lines (data not shown and Supplemental Figure 1); however, 72-h CM of 8 of 15 neuroblastoma cell lines stimulated monocytes to secrete IL-6 after 24 h (Fig. 2b). IL-10 was minimally secreted by CHLA-255-Fluc cells or monocytes alone but was secreted by co-cultured cells (Supplemental Figure 1). CHLA-255-Fluc cells and monocytes alone or co-cultured secreted <25 pg/ml of IFNγ, IL-2, IL-15, IL-12p70 (functional heterodimer of IL-12p35 and IL-12p40 subunits) (Supplemental Figure 1). Thus, the profile of cytokines released by neuroblastoma cell lines alone, by monocytes cultured in tumor cell CM, or by neuroblastoma/monocyte co-cultures is comparable to the profile of cytokine gene expression in primary untreated tumors.

### Suppression of IL-2 induction of NK cell cytotoxicity and IFNγ secretion by IL-6 and TGFβ1 in neuroblastoma/monocyte-conditioned medium is prevented by lenalidomide

Based upon the release of TGFβ1 by tumor cells and of IL-6 and TGFβ1 by monocytes cultured in tumor cell CM and upon the possibility that these cytokines could suppress activation of NK cells [5, 7], we determined the effect of neuroblastoma/monocyte co-culture CM on IL-2 activation of purified NK cells using cytotoxicity and IFNγ secretion as end points (Fig. 2c–e). Indeed, 50 % CM (volume/volume), which contained IL-6 (560 pg/ml) and TGFβ1 (410 pg/ml), significantly suppressed the activation of NK cells for direct cytotoxicity and ADCC and for the secretion of IFNγ (Fig. 2c–e). Although the neuroblastoma/monocyte CM contained IL-6 and TGFβ1, these cytokines were increased further in cultures of NK cells and IL-2 (Fig. 2e).

Lenalidomide has been reported to activate NK cells [14, 15], and we confirmed this using clinically achievable doses (Supplemental Figure 2). Based upon this strong activity, we hypothesized that neuroblastoma/monocyte suppression of NK cell activation could be overcome by lenalidomide. As shown in Fig. 2c–e, inclusion of lenalidomide in cultures containing 50 % neuroblastoma/monocyte CM prevented the suppression of IL-2-induced direct cytotoxicity, ADCC, and IFNγ secretion. Inclusion of lenalidomide also suppressed CM induction of NK cell secretion of IL-6 and TGFβ1 (Fig. 2e).

To determine whether IL-6 and TGFβ1 were responsible for the suppression of NK cell cytotoxicity and ADCC, they were depleted from the neuroblastoma/monocyte CM with anti-cytokine antibodies (Supplemental Figure 3). Combined depletion of IL-6 and TGFβ1 from the CM with anti-cytokine antibodies removed inhibition of IL-2 activation of NK cells for direct cytotoxicity and ADCC (Supplemental Figure 3). These data along with the gene expression profile of neuroblastoma tumors and cytokines released by neuroblastoma and monocyte co-cultures suggest that neuroblastoma/monocyte interactions generate a cytokine milieu that suppresses NK cell anti-tumor functions.

### Suppression of IL-2 induction of NK cell cytotoxicity and IFNγ secretion by recombinant IL-6 and TGFβ1 is prevented by lenalidomide

**Fig. 3:**
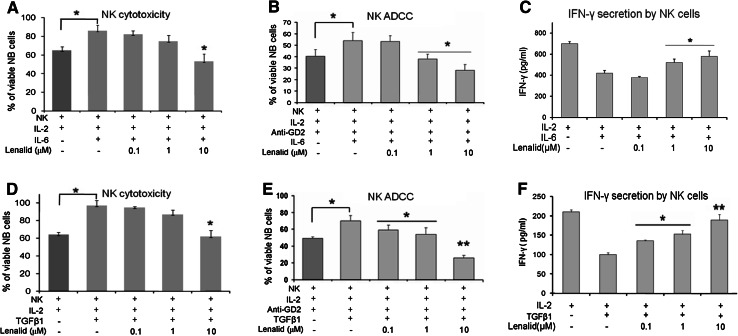
Suppression of IL-2 induction of NK cell direct cytotoxicity, ADCC, and IFNγ secretion by IL-6 and TGFβ1 is prevented by lenalidomide. **a**, **b** Purified NK cells (1 × 10^4^ cells/0.1 ml/well) were cultured for 72 h with IL-2 alone (10 ng/ml) or with added IL-6 (10 ng/ml) and lenalidomide as indicated, and then NK cell-mediated cytotoxicity and ADCC (*E*:*T* ratio = 2:1) with ch14.18 (0.1 μg/ml) were quantified after 6 h of co-culture with CHLA-255-Fluc cells with the calcein-AM/DIMSCAN assay (mean ± SD for 8 replicate cultures for each condition). Confirmatory results were obtained from 3 additional experiments. **c** NK cells (5 × 10^5^/ml) were cultured for 24 h with IL-2 alone (10 ng/ml) or with added IL-6 (10 ng/ml) and lenalidomide as indicated, and then IFNγ was quantified in the medium by ELISA (mean ± SD for 3 replicate cultures for each condition). Confirmatory results were obtained with 2 additional experiments. **d**, **e** NK cells (1 × 10^4^ cells/0.1 ml/well) were cultured for 72 h with IL-2 alone (10 ng/ml) or with added TGFβ1 (10 ng/ml) and lenalidomide as indicated, and then NK cell-mediated cytotoxicity and ADCC (*E*:*T* ratio = 2:1) with ch14.18 (0.1 μg/ml) were quantified after 6 h of co-culture with CHLA-255-Fluc cells with the calcein-AM/DIMSCAN assay (mean ± SD for 8 replicate cultures for each condition). Confirmatory results were obtained from 3 additional experiments. **f** NK cells (5 × 10^5^ cells/ml) were cultured for 24 h with IL-2 alone (10 ng/ml) or with added TGFβ1 (10 ng/ml) and lenalidomide as indicated, and then IFNγ was quantified in the medium by ELISA (mean ± SD for 3 replicate cultures for each condition). Confirmatory results were obtained with 1 additional experiment. The *t* test *P* values, no lenalidomide versus lenalidomide *P < 0.05; **P < 0.01

Since the suppression of NK cell activation by neuroblastoma/monocyte CM was likely due to IL-6 [5] and/or TGFβ1 [7], we next tested these cytokines alone and with lenalidomide to determine the effects upon NK cell activation. Purified NK cells were cultured for 72 h with IL-2 and IL-6 or TGFβ1 without or with lenalidomide and then tested for direct cytotoxicity, ADCC, and IFNγ secretion. NK cells cultured with IL-6 or TGFβ1 mediated less direct cytotoxicity and ADCC and secreted less IFNγ than those cultured with IL-2 alone (Fig. 3). However, the suppression of NK cell cytotoxicity functions by these cytokines was prevented by the addition of lenalidomide to the cultures (Fig. 3). Similarly, the suppression of IFNγ secretion by NK cells was largely overcome by lenalidomide (Fig. 3). IL-6, similar to the neuroblastoma/monocyte CM (Fig. 2), significantly increased NK cell secretion of IL-6, whereas lenalidomide prevented this effect (data not shown). TGFβ1, in contrast, decreased NK cell secretion of IL-6, and this was not altered by lenalidomide (data not shown). Thus, anti-tumor functions of NK cells are suppressed by IL-6 and TGFβ1, and this is largely prevented by lenalidomide.

### Suppression of NK cell functions contributing to cytotoxicity by recombinant IL-6 and TGFβ1 is prevented by lenalidomide

**Fig. 4:**
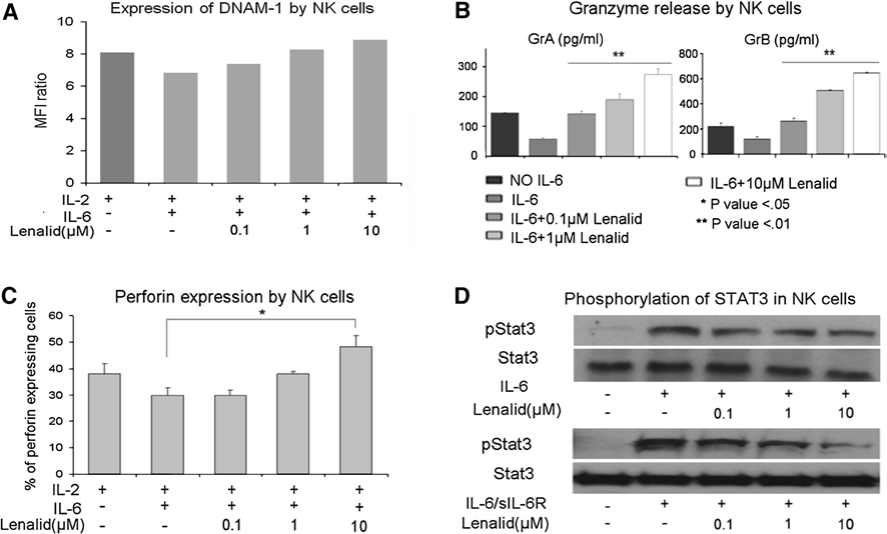
Suppression of NK cell cytotoxicity-related functions and activation of STAT3 by IL-6 is prevented by lenalidomide. **a** Purified NK cells (5 × 10^5^ cells/ml) were cultured for 72 h with IL-2 alone (10 ng/ml) or with addition of IL-6 (10 ng/ml) and lenalidomide as indicated, and then the expression of DNAM-1 was evaluated with flow cytometry. A similar pattern of little or no effect was observed for NKG2D, NKp46, CD16, CD56, and CD107a (data not shown). **b** NK cells (5 × 10^5^ cells/ml) were cultured for 72 h with IL-2 alone (10 ng/ml) or with added IL-6 (10 ng/ml) and lenalidomide as indicated, and then granzymes A and B were quantified in the CM with the CBA assay (mean ± SD for 3 replicate cultures for each condition). Confirmatory results were obtained with 2 additional experiments. **c** NK cells (5 × 10^5^ cells/ml) were cultured for 72 h with IL-2 alone (10 ng/ml) or with added IL-6 (10 ng/ml) and lenalidomide, and then intracellular perforin was analyzed by flow cytometry. The percent of NK cells expressing perforin in the different culture conditions is shown (mean ± SD for 3 replicate cultures for each condition). Confirmatory results were obtained with 2 additional experiments. **d** NK cells were preincubated (20 min) with lenalidomide at the indicated concentrations, treated with IL-6 (10 ng/ml) or IL-6 plus sIL-6R (25 ng/ml) for 30 min, and then lysed for the analysis of phosphorylated STAT3 by Western blotting. Densitometry confirmed that lenalidomide inhibited phosphorylation of STAT3. Confirmatory results were obtained with 1 additional experiment. The *t* test *P* values *P < 0.05; **P < 0.01

Effects of IL-6 and TGFβ1 on NK cell functions that contribute to cytotoxicity without and with lenalidomide were determined. When IL-6 was added to NK cells cultured for 72 h with IL-2, it did not affect the expression of cell surface NKG2D, DNAM-1, NKp46, CD16, adhesion molecule CD56, or degranulation marker CD107a (Fig. 4a and data not shown). However, IL-6 did suppress the release of granzymes A and B and expression of perforin by purified NK cells (Fig. 4b, c). Lenalidomide, in the presence of IL-6, did not increase the expression of the evaluated cell surface molecules with exception of CD56 (Fig. 4a and data not shown), but it did prevent the suppression of granzymes A and B release and of perforin expression (Fig. 4b, c).

**Fig. 5:**
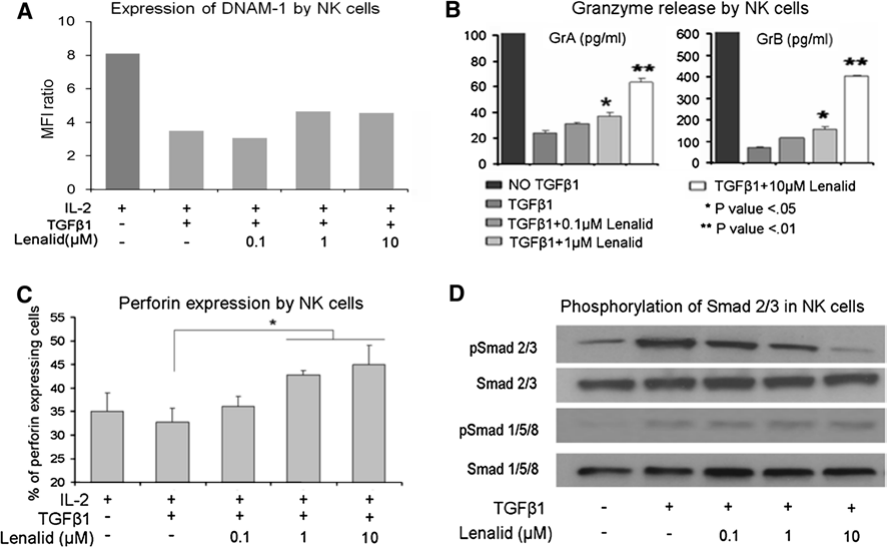
Suppression of NK cell cytotoxicity-related functions and activation of SMAD2/3 by TGFβ1 is prevented by lenalidomide. **a** NK cells (5 × 10^5^ cells/ml) were cultured for 72 h with IL-2 alone (10 ng/ml) or with added TGFβ1 (10 ng/ml) and lenalidomide as indicated, and then the expression of DNAM-1 was evaluated with flow cytometry. A similar pattern of suppression was observed for NKG2D, NKp46, CD16, CD56, and CD107a (Supplemental Figure 4). **b** NK cells (5 × 10^5^ cells/ml) were cultured for 72 h with IL-2 alone (10 ng/ml) or with added TGFβ1 (10 ng/ml) and lenalidomide as indicated, and then granzymes A and B were quantified in the CM with the CBA assay (mean ± SD for 3 replicate cultures for each condition). Confirmatory results were obtained from 2 additional experiments. **c** NK cells (5 × 10^5^ cells/ml) were cultured for 72 h with IL-2 alone (10 ng/ml) or with added TGFβ1 (10 ng/ml) and lenalidomide, and then intracellular perforin was analyzed by flow cytometry. The percent of NK cells expressing perforin in the different culture conditions is shown (mean ± SD for 3 replicate experiments for each condition). **d** NK cells were preincubated (20 min) with lenalidomide at the indicated concentrations, treated with TGFβ1 (10 ng/ml) for 20 min, and then lysed for the analysis of phosphorylated SMAD2/3 by Western blotting. Densitometry confirmed that lenalidomide inhibited phosphorylation of SMAD2/3. Confirmatory results were obtained with 1 additional experiment. The *t* test *P* values *P < 0.05; **P < 0.01

When TGFβ1 was added to NK cells cultured for 72 h with IL-2, it suppressed the expression of cell surface NKG2D, DNAM-1, NKp46, CD16, CD56, and CD107a as well as release of granzymes A and B and expression of perforin (Fig. 5a–c; Supplemental Figure 4). Lenalidomide did not prevent TGFβ1 suppression of NKG2D, DNAM-1, NKp46, or CD16 but did prevent its inhibitory effect upon expression of CD56 and CD107a, release of granzymes A and B, and expression of perforin (Fig. 5a–c; Supplemental Figure 4).

### IL-6-induced STAT3 phosphorylation and TGFβ1-induced SMAD2/3 phosphorylation in NK cells are inhibited by lenalidomide

STAT3 was phosphorylated in purified NK cells following stimulation with IL-6 alone or combined with sIL-6R, as assessed by Western blotting (Fig. 4d). To determine whether lenalidomide could block IL-6 stimulation of STAT3 phosphorylation, NK cells were treated for 20 min with lenalidomide (0.1, 1, and 10 μM), followed by treatment for another 30 min with IL-6 (10 ng/ml) alone or with sIL-6R (25 ng/ml). Lenalidomide significantly inhibited IL-6- and IL-6/sIL-6R-induced STAT3 phosphorylation in NK cells (Fig. 4d). This suggests that the inhibition of STAT3 phosphorylation by lenalidomide may be one mechanism, whereby it blocks the inhibitory effect of IL-6 on NK cell activation by IL-2.

To test the effect of lenalidomide upon TGFβ1-induced signaling, NK cells were treated for 20 min with lenalidomide (0.1, 1, and 10 μM) followed by treatment for another 20 min with TGFβ1 (10 ng/ml). Western blotting showed that TGFβ1 induced the phosphorylation of SMAD2/3 and SMAD1/5/8 and that lenalidomide inhibited the phosphorylation of SMAD2/3 but not of SMAD1/5/8 (Fig. 5d). The inhibitory effect of lenalidomide was further confirmed by fluorescence microscopy with anti-phospho-SMAD2/3, which demonstrated markedly decreased immunostaining and nuclear translocation (Supplemental Figure 5A and 5B).

### NK cell anti-neuroblastoma activity with anti-GD2 mAb ch14.18 in NOD/SCID mice is enhanced by lenalidomide

**Fig. 6:**
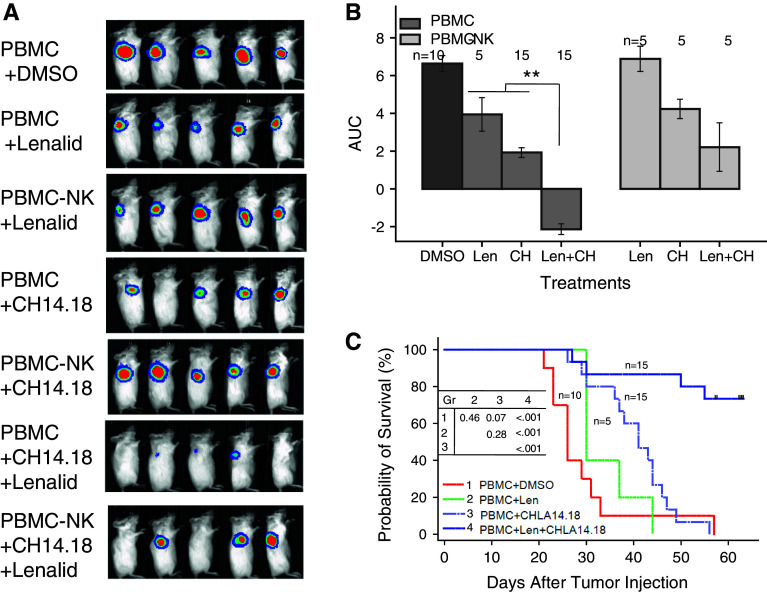
NK cells among PBMC that are co-injected with neuroblastoma cells into NOD/SCID mice are most effective when mice are treated with both lenalidomide and ch14.18. CHLA-255-Fluc neuroblastoma cells (10^6^) and PBMC (0.25 × 10^6^; CD3+ 75.3 %; CD3–CD56+ CD16+ 2.7 %) or NK cell depleted PBMC (PBMC-NK) (0.25 × 10^6^; CD3+ 82.6 %; CD3–CD56+ CD16+ 0.02 %) were co-injected into NOD/SCID mice near the right and left shoulders in 25 % (v/v) BD Matrigel^®^ Matrix Growth Factor Reduced on day 0. Treatment began on day 1 (after imaging and randomization) with lenalidomide alone (50 mg/kg/d intraperitoneal days 1–9 and 12–16), ch14.18 alone (15 μg/mouse intravenous days 3, 5, 8, and 12), or the combination of lenalidomide and ch14.18 using the same doses and schedules. **a** Bioluminescence images at day 8 for the different groups are shown. **b** Growth of neuroblastoma tumors. Bioluminescence imaging measurements were taken on the *left* and *right sides* of each mouse and were collected 1, 8, and 15 days after injection of tumor cells for calculation of area under the curve (AUC) for each group. Data for mice receiving PBMC (two experiments) or PBMC-NK (one of the two experiments) are shown. PBMC mice that were treated with both lenalidomide and ch14.18 had smaller AUC compared to untreated (*P* < 0.001), lenalidomide alone (*P* = 0.007), or ch14.18 alone (*P* = 0.011). PBMC-NK mice that were treated with both lenalidomide and ch14.18 did not have smaller AUC compared to lenalidomide or ch14.18 alone. Tumor growth in mice receiving PBMC versus PBMC-NK was no different with lenalidomide or ch14.18 alone treatment but was less for the PBMC group treated with the combination of lenalidomide and ch14.18 (*P* = 0.006). Overall, there was a significant reduction in treatment efficacy as measured by AUC with PBMC-NK versus PBMC (*P* = 0.017). **c** Survival of mice in different groups. Survival time, which is defined as the days from tumor cell injection until the mouse is killed due to tumor size or the end of the experiment (day 56), was greater for PBMC mice that were treated with both lenalidomide and ch14.18 than for untreated (*P* < 0.0001), lenalidomide-treated (*P* < 0.0001) or ch14.18-treated (*P* < 0.0001) groups. PBMC-NK mice that were treated with both lenalidomide and ch14.18 survived longer than those treated with lenalidomide (*P* = 0.03) or ch14.18 (*P* = 0.04) alone. Overall, there was a significant reduction in treatment efficacy as measured by survival with PBMC-NK versus PBMC (*P* = 0.048) (Supplemental Figure 7). Images in Fig. 6a are from one experiment, whereas data in Figs. 6b and c are combined from two experiments

A NOD/SCID mouse model of local disease was used in which CHLA-255-Fluc neuroblastoma cells (10^6^) were co-injected with PBMC (0.25 × 10^6^) in 25 % (v/v) BD Matrigel™ Matrix Growth Factor Reduced, which includes TGFβ1. In this model, the SMAD2/3 and STAT3 signaling pathways are activated in tumor cells as shown using CHLA-255 SMAD2/3 and STAT3 Fluc reporter cell lines, and activation is inhibited by lenalidomide (Supplementary Figure 6A and 6B). Based on these in vivo data and our in vitro experiments, we reasoned that the pathways also could be activated in NK cells among the PBMC in vivo but that lenalidomide could overcome this and enhance NK cell anti-tumor functions. Mice were treated with lenalidomide alone, ch14.18 alone, or both together (Fig. 6a). After 14 days of treatment, lenalidomide combined with ch14.18 most effectively suppressed tumor cell growth (AUC) compared to untreated (*P* < 0.001), lenalidomide alone (*P* = 0.007), or ch14.18 alone (*P* = 0.011)-treated mice (Fig. 6B). Survival time was also longer for the lenalidomide plus ch14.18-treated group than for untreated (*P* < 0.0001), lenalidomide-treated (*P* < 0.0001) or ch14.18-treated (*P* < 0.0001) groups (Fig. 6c). When mice receiving NK-depleted PBMC were treated with lenalidomide plus ch14.18, they had greater tumor growth (*P* = 0.05) and poorer survival (*P* = 0.006) than the PBMC group (Fig. 6b and Supplemental Figure 7). Tumor growth was no different for these two groups treated with lenalidomide or ch14.18 alone, and survival was no different for lenalidomide but was marginally poorer for the NK-depleted group and ch14.18 alone-treated group (*P* = 0.075) (Supplemental Figure 7). Together, these data indicate that lenalidomide enhances survival of mice treated with ch14.18 and that this in vivo effect involves NK cells.

## Discussion

This study demonstrates the potential immunosuppressive impact of neuroblastoma cells interacting with monocytes/macrophages in the tumor microenvironment upon anti-tumor cell functions of NK cells alone or combined with the anti-neuroblastoma GD2 mAb ch14.18. IL-6, which is secreted by mononuclear phagocytes in response to neuroblastoma cells, and TGFβ1, which is secreted by both tumor cells and mononuclear phagocytes in response to neuroblastoma cells, suppress the activation of NK cells by IL-2. We show for the first time that lenalidomide, an immune-modulating drug that enhances IL-2 activation of NK cells, prevents suppressive effects of both IL-6 and TGFβ1, likely by inhibiting the induction of their respective signaling pathways, i.e., STAT3 for IL-6 and SMAD2/3 for TGFβ1. Maximal suppression of neuroblastoma tumor growth in NOD/SCID mice by NK cells in a microenvironment in which tumor cell STAT3 and SMAD2/3 pathways are active occurs when mice are treated with both lenalidomide and ch14.18. These results suggest that therapy with antibodies that mediate ADCC with NK cells may be improved by the addition of lenalidomide, which both activates NK cells and prevents their suppression by IL-6 and TGFβ1 in the microenvironment.

We compared gene expression of neuroblastoma tumors and cell lines using a TLDA assay and found that tumors have higher expression of TAM-associated genes as well as of IL-6 and IL-10 than cell lines. TGFβ1 was strongly expressed by both tumors and cell lines, suggesting that tumor cells are a major source of this cytokine. Expression of IFNγ, IL-2, IL-12, and IL-15 was low in both tumors and cell lines. Quantifying cytokines released by neuroblastoma cell lines and cell line/monocyte co-cultures showed that the cytokine profiles of CM from co-cultures were similar to the gene expression signature of tumors. These data support the conclusion that neuroblastoma tumors are rich in cytokines that are potentially immunosuppressive (IL-6, IL-10, and TGFβ1) but not in cytokines that support NK cell proliferation, differentiation, and activation (IL-2, IL-15, IL-12).

Neuroblastoma cell/monocyte co-cultures generated CM that contained IL-6 and TGFβ1 and that suppressed IL-2 activation of NK cells for direct cytotoxicity, ADCC, and IFNγ secretion. Depletion of IL-6 and TGFβ1 from CM by combining antibodies against both cytokines removed suppression. We discovered that lenalidomide, at a clinically achievable concentration [16, 32], prevented the suppression of IL-2-mediated activation of NK cell cytotoxicity, ADCC, and IFNγ secretion by neuroblastoma/monocyte CM. Recombinant IL-6 and TGFβ1 were used at functionally effective doses to model the neuroblastoma/monocyte generated molecules to study the effects of these cytokines individually without or with lenalidomide upon NK cell activation.

We demonstrated for the first time that IL-6, likely via activation of the STAT3 signaling pathway, suppresses IL-2 induction of human NK cell direct cytotoxicity and ADCC, which is accompanied by decreased release of granzymes A and B and expression of perforin but not by altered expression of NKG2D, DNAM-1, NKp46, and CD16. We also show that IL-6, similar to the neuroblastoma/monocyte CM, significantly suppresses release of IFNγ by IL-2-stimulated NK cells. As with experiments testing neuroblastoma/monocyte CM, we discovered that lenalidomide, at clinically achievable concentrations [16, 32], prevented IL-6 suppression of IL-2-mediated activation of NK cell cytotoxicity, ADCC, and IFNγ secretion.

TGFβ1 has previously been shown to suppress, via SMAD3, IL-2 activation of NK cells for ADCC and IL-2 or IL-12 plus CD16 activation for IFNγ secretion [7]. TGFβ1 also has been reported to suppress the expression of NKG2D and CD16 [10–13]. Our data confirm that TGFβ1 activates SMAD2/3 and inhibits NK cell expression of NKG2D and CD16 and mediation of ADCC. We extend previous information by showing that the expression of DNAM-1, NKp46, CD56, CD107a and perforin, and the release of granzymes A and B are decreased by TGFβ1. IL-2-stimulated NK cell secretion of IFNγ was decreased by TGFβ1. Suppression of direct cytotoxicity, ADCC, and IFNγ secretion by TGFβ1 was also prevented by lenalidomide.

The mechanisms underlying the effects of lenalidomide appear to be at least partially due to the inhibition of STAT3 and SMAD2/3 phosphorylation by IL-6 and TGFβ1, respectively. Lenalidomide has previously been reported to inhibit phosphorylation of STAT3 in a multiple myeloma cell line, and this was enhanced by the addition of simvastatin [33]. However, lenalidomide has not been reported to inhibit phosphorylation of STAT3 for any other cell type. Attribution of changes in NK cell effector functions to the activation of the STAT3 pathway is supported by the observation that genetic ablation of STAT3 in hematopoietic cells of mice bearing B16 melanoma resulted in greater cytotoxicity of spleen NK cells compared to those from tumor-bearing wild-type mice [6]. Inhibition of the phosphorylation of SMAD2/3 by lenalidomide has not been previously reported for any cell type. Using STAT3 and SMAD2/3 reporter neuroblastoma cells, we showed that reporter activity was inhibited when treatment with lenalidomide was begun 72 h after injection of tumor cells into NOD/SCID mice, which suggest that lenalidomide may block ongoing phosphorylation and so contribute to a decrease in the level of activation. Although the mechanisms whereby lenalidomide inhibits the activation of these pathways remain to be determined, our findings suggest that it may enhance the activity of agents that specifically target these pathways.

Using a model in which human PBMC and neuroblastoma cells are co-injected into NOD/SCID mice, we showed that lenalidomide increases the in vivo anti-tumor cell activity of mAb ch14.18. Human NK cells appear to be the main effectors with combined lenalidomide and ch14.18 treatment because tumor growth was less and survival was longer for mice receiving PBMC versus NK-depleted PBMC. Another study that used Raji lymphoma cells growing in SCID mice demonstrated that murine NK cells can mediate the anti-tumor effect of rituximab combined with pomalidomide or lenalidomide [18]. It is unlikely that murine NK cells contributed significantly to the anti-tumor effect of lenalidomide combined with mAb ch14.18 in our experiments since we depleted them from NOD/SCID mice with an anti-IL-2Rβ/CD122 mAb [26] before neuroblastoma and PBMC co-injection.

Clinical trials of lenalidomide in children and adults have demonstrated immunological effects that would be expected to enhance ADCC in vivo without the significant toxicities observed with the combination of ch14.18, IL-2, and GM-CSF [1, 16, 17, 32]. Our preclinical data and the immunological effects observed in clinical trials of lenalidomide [16, 17] suggest that the combination of lenalidomide and ch14.18 will be active against neuroblastoma in patients. Of importance, the toxicity of such a regimen may be less than the current standard regimen of ch14.18, IL-2, and GM-CSF.

In summary, our study demonstrates that the microenvironmental milieu of neuroblastoma cells includes IL-6 and TGFβ1, which suppress IL-2 activation of NK cells for direct cytotoxicity, ADCC, and IFNγ secretion. Our finding that lenalidomide both enhances activation and overcomes the suppression of NK cell anti-tumor functions suggests that it may improve efficacy of treatment with anti-tumor cell antibodies such as ch14.18.

### Electronic supplementary material

Below is the link to the electronic supplementary material.

Supplementary material 1 (PDF 762 kb)

## References

[1] Yu AL, Gilman AL, Ozkaynak MF, London WB, Kreissman SG, Chen HX, Smith M, Anderson B, Villablanca JG, Matthay KK, Shimada H, Grupp SA, Seeger R, Reynolds CP, Buxton A, Reisfeld RA, Gillies SD, Cohn SL, Maris JM, Sondel PM (2010). Anti-GD2 antibody with GM-CSF, interleukin-2, and isotretinoin for neuroblastoma. N Engl J Med.

[2] Asgharzadeh S, Pique-Regi R, Sposto R, Wang H, Yang Y, Shimada H, Matthay K, Buckley J, Ortega A, Seeger RC (2006). Prognostic significance of gene expression profiles of metastatic neuroblastomas lacking MYCN gene amplification. J Natl Cancer Inst.

[3] Song L, Asgharzadeh S, Salo J, Engell K, Wu HW, Sposto R, Ara T, Silverman AM, DeClerck YA, Seeger RC, Metelitsa LS (2009). Valpha24-invariant NKT cells mediate antitumor activity via killing of tumor-associated macrophages. J Clin Invest.

[4] Asgharzadeh S, Salo JA, Ji L, Oberthuer A, Fischer M, Berthold F, Hadjidaniel M, Liu CW, Metelitsa LS, Pique-Regi R, Wakamatsu P, Villablanca JG, Kreissman SG, Matthay KK, Shimada H, London WB, Sposto R, Seeger RC (2012). Clinical significance of tumor-associated inflammatory cells in metastatic neuroblastoma. J Clin Oncol.

[5] Scheid C, Young R, McDermott R, Fitzsimmons L, Scarffe JH, Stern PL (1994). Immune function of patients receiving recombinant human interleukin-6 (IL-6) in a phase I clinical study: induction of C-reactive protein and IgE and inhibition of natural killer and lymphokine-activated killer cell activity. Cancer Immunol Immunother.

[6] Kortylewski M, Kujawski M, Wang T, Wei S, Zhang S, Pilon-Thomas S, Niu G, Kay H, Mule J, Kerr WG, Jove R, Pardoll D, Yu H (2005). Inhibiting Stat3 signaling in the hematopoietic system elicits multicomponent antitumor immunity. Nat Med.

[7] Trotta R, Col JD, Yu J, Ciarlariello D, Thomas B, Zhang X, Allard J, Wei M, Mao H, Byrd JC, Perrotti D, Caligiuri MA (2008). TGF-beta utilizes SMAD3 to inhibit CD16-mediated IFN-gamma production and antibody-dependent cellular cytotoxicity in human NK cells. J Immunol.

[8] Bellone G, Aste-Amezaga M, Trinchieri G, Rodeck U (1995). Regulation of NK cell functions by TGF-beta 1. J Immunol.

[9] Yu J, Wei M, Becknell B, Trotta R, Liu S, Boyd Z, Jaung MS, Blaser BW, Sun J, Benson DM, Mao H, Yokohama A, Bhatt D, Shen L, Davuluri R, Weinstein M, Marcucci G, Caligiuri MA (2006). Pro- and antiinflammatory cytokine signaling: reciprocal antagonism regulates interferon-gamma production by human natural killer cells. Immunity.

[10] Keskin DB, Allan DS, Rybalov B, Andzelm MM, Stern JN, Kopcow HD, Koopman LA, Strominger JL (2007). TGFbeta promotes conversion of CD16 + peripheral blood NK cells into CD16- NK cells with similarities to decidual NK cells. Proc Natl Acad Sci USA.

[11] Allan DS, Rybalov B, Awong G, Zuniga-Pflucker JC, Kopcow HD, Carlyle JR, Strominger JL (2010). TGF-beta affects development and differentiation of human natural killer cell subsets. Eur J Immunol.

[12] Friese MA, Wischhusen J, Wick W, Weiler M, Eisele G, Steinle A, Weller M (2004). RNA interference targeting transforming growth factor-beta enhances NKG2D-mediated antiglioma immune response, inhibits glioma cell migration and invasiveness, and abrogates tumorigenicity in vivo. Cancer Res.

[13] Lee JC, Lee KM, Kim DW, Heo DS (2004). Elevated TGF-beta1 secretion and down-modulation of NKG2D underlies impaired NK cytotoxicity in cancer patients. J Immunol.

[14] Bartlett JB, Dredge K, Dalgleish AG (2004). The evolution of thalidomide and its IMiD derivatives as anticancer agents. Nat Rev Cancer.

[15] Hayashi T, Hideshima T, Akiyama M, Podar K, Yasui H, Raje N, Kumar S, Chauhan D, Treon SP, Richardson P, Anderson KC (2005). Molecular mechanisms whereby immunomodulatory drugs activate natural killer cells: clinical application. Br J Haematol.

[16] Berg SL, Cairo MS, Russell H, Ayello J, Ingle AM, Lau H, Chen N, Adamson PC, Blaney SM (2011). Safety, pharmacokinetics, and immunomodulatory effects of lenalidomide in children and adolescents with relapsed/refractory solid tumors or myelodysplastic syndrome: a Children’s Oncology Group Phase I Consortium report. J Clin Oncol.

[17] Bartlett JB, Michael A, Clarke IA, Dredge K, Nicholson S, Kristeleit H, Polychronis A, Pandha H, Muller GW, Stirling DI, Zeldis J, Dalgleish AG (2004). Phase I study to determine the safety, tolerability and immunostimulatory activity of thalidomide analogue CC-5013 in patients with metastatic malignant melanoma and other advanced cancers. Br J Cancer.

[18] Hernandez-Ilizaliturri FJ, Reddy N, Holkova B, Ottman E, Czuczman MS (2005). Immunomodulatory drug CC-5013 or CC-4047 and rituximab enhance antitumor activity in a severe combined immunodeficient mouse lymphoma model. Clin Cancer Res.

[19] Wu L, Adams M, Carter T, Chen R, Muller G, Stirling D, Schafer P, Bartlett JB (2008). lenalidomide enhances natural killer cell and monocyte-mediated antibody-dependent cellular cytotoxicity of rituximab-treated CD20+ tumor cells. Clin Cancer Res.

[20] Zhang L, Qian Z, Cai Z, Sun L, Wang H, Bartlett JB, Yi Q, Wang M (2009). Synergistic antitumor effects of lenalidomide and rituximab on mantle cell lymphoma in vitro and in vivo. Am J Hematol.

[21] Tai YT, Li XF, Catley L, Coffey R, Breitkreutz I, Bae J, Song W, Podar K, Hideshima T, Chauhan D, Schlossman R, Richardson P, Treon SP, Grewal IS, Munshi NC, Anderson KC (2005). Immunomodulatory drug lenalidomide (CC-5013, IMiD3) augments anti-CD40 SGN-40-induced cytotoxicity in human multiple myeloma: clinical implications. Cancer Res.

[22] Lapalombella R, Gowda A, Joshi T, Mehter N, Cheney C, Lehman A, Chen CS, Johnson AJ, Caligiuri MA, Tridandapani S, Muthusamy N, Byrd JC (2009). The humanized CD40 antibody SGN-40 demonstrates pre-clinical activity that is enhanced by lenalidomide in chronic lymphocytic leukaemia. Br J Haematol.

[23] Wu L, Parton A, Lu L, Adams M, Schafer P, Bartlett JB (2011). Lenalidomide enhances antibody-dependent cellular cytotoxicity of solid tumor cells in vitro: influence of host immune and tumor markers. Cancer Immunol Immunother.

[24] Xu Y, Li J, Ferguson GD, Mercurio F, Khambatta G, Morrison L, Lopez-Girona A, Corral LG, Webb DR, Bennett BL, Xie W (2009). Immunomodulatory drugs reorganize cytoskeleton by modulating Rho GTPases. Blood.

[25] Chen RL, Reynolds CP, Seeger RC (2000). Neutrophils are cytotoxic and growth-inhibiting for neuroblastoma cells with an anti-GD2 antibody but, without cytotoxicity, can be growth-stimulating. Cancer Immunol Immunother.

[26] Tanaka T, Kitamura F, Nagasaka Y, Kuida K, Suwa H, Miyasaka M (1993). Selective long-term elimination of natural killer cells in vivo by an anti-interleukin 2 receptor beta chain monoclonal antibody in mice. J Exp Med.

[27] Waldmann TA (2006). The biology of interleukin-2 and interleukin-15: implications for cancer therapy and vaccine design. Nat Rev Immunol.

[28] Seeger RC, Rayner SA, Banerjee A, Chung H, Laug WE, Neustein HB, Benedict WF (1977). Morphology, growth, chromosomal pattern and fibrinolytic activity of two new human neuroblastoma cell lines. Cancer Res.

[29] Seeger RC, Danon YL, Rayner SA, Hoover F (1982). Definition of a Thy-1 determinant on human neuroblastoma, glioma, sarcoma, and teratoma cells with a monoclonal antibody. J Immunol.

[30] Reynolds CP, Biedler JL, Spengler BA, Reynolds DA, Ross RA, Frenkel EP, Smith RG (1986). Characterization of human neuroblastoma cell lines established before and after therapy. J Natl Cancer Inst.

[31] Keshelava N, Seeger RC, Groshen S, Reynolds CP (1998). Drug resistance patterns of human neuroblastoma cell lines derived from patients at different phases of therapy. Cancer Res.

[32] Warren KE, Goldman S, Pollack IF, Fangusaro J, Schaiquevich P, Stewart CF, Wallace D, Blaney SM, Packer R, Macdonald T, Jakacki R, Boyett JM, Kun LE (2011). Phase I trial of lenalidomide in pediatric patients with recurrent, refractory, or progressive primary CNS tumors: pediatric Brain Tumor Consortium study PBTC-018. J Clin Oncol.

[33] van der Spek E, Bloem AC, Lokhorst HM, van Kessel B, Bogers-Boer L, van de Donk NW (2009). Inhibition of the mevalonate pathway potentiates the effects of lenalidomide in myeloma. Leuk Res.

