# Supplementary material for: Lenalidomide overcomes suppression of human natural killer cell anti-tumor functions by neuroblastoma microenvironment-associated IL-6 and TGFβ1
Source: Cancer Immunol Immunother. 2013 Aug 27;62(10):1637–48. doi: 10.1007/s00262-013-1466-y (PMC3907789; doi:10.1007/s00262-013-1466-y)
Supplement: Supplementary file 1 — Supplementary material 1 (PDF 762 kb) [file 262_2013_1466_MOESM1_ESM.pdf]

Supplemental Methods and Figures for Xu et al.

Methods

Reagents

Iscove's Modified Dulbecco's Medium (IMDM) and fetal bovine serum (FBS) were from Invitrogen, and Dulbecco's PBS without calcium or magnesium was from MediaTech. Chemicals were from Sigma-Aldrich unless otherwise specified. Recombinant human TGFβ1 and IL-6 were purchased from R&D systems. TGFβ1 and IFNγ ELISA kits were from eBioscience, and the IL-6 ELISA kit was from R&D systems. Cytometric Bead Array (CBA) kits were from BD Biosciences, and Luminex® Milliplex® multi-analyte panels were from Millipore.

Antibodies used for flow cytometry and Western blotting are as follows. FITC-labeled anti-DNAM-1 (clone DX11), APC-labeled anti-CD107a (clone H4A3), and APC-H7-labled anti-CD16 (clone 3G8) were from BD Biosciences. PE-labeled anti-NKG2D (clone 1D11), Pacific Blue-labeled anti-NKp46 (clone 9E2), Alexa Fluor 700-labeled anti-CD56 (clone HCD56), and PE-Cy7-labeled anti-CD3 (clone SK7) antibodies were from BioLegend. Pacific Orange-labeled anti-CD14 (clone Tuk4) was from Life Technologies. Anti-GD2 mouse antibody 14G2a was purified in our laboratory from the hybridoma culture medium and labeled with FITC. Human FcR blocking agent was from Miltenyi Biotec. Anti-human STAT3, phospho-STAT3(Tyr705) SMAD2/3, phospho-SMAD2(S465/S467)/SMAD3(S423/S425), SMAD1/5/8, phospho-Smad1(Ser463/465)/Smad5(Ser463/465)/Smad8(Ser426/428), and β-actin were from Cell Signaling.

Neuroblastoma cell line transductions

CHLA-255 reporter stable cell lines were established according to the manufacturer's instructions by transducing CHLA255 cells expressing hRluc with the Cignal Lenti Fluc reporters. The Cignal Lenti SMAD2/3 and STAT3 firefly luciferase reporters for monitoring transcriptional activity in ready-to-transduce lentiviral particles were from SABiosciences.

Puromycin (5μg/ml) was applied to the transduced cells to generate hRluc reporter stable CHLA255 cell lines.

TaqMan® Low-Density Array (TLDA) assays

Total RNA from frozen sections of primary neuroblastoma tumors and from neuroblastoma cell lines was previously isolated for microarray analysis using TRIzol reagent at Children's Hospital Los Angeles [2]. Custom and predesigned human immune TLDA Panels were used in a reverse transcriptase polymerase chain reaction (RT-PCR) process employing the ABI Prism® 7900 HT Sequence Detection System (Applied Biosystems). A total of 100 μl of reaction mixture with 50 μl cDNA template (500 ng) and an equal volume of TaqMan® universal master mix (Applied Biosystems) were added to each port of the TLDA card after gentle vortex mixing. Thermal cycler conditions were as follows: 2 minutes at 50°C, 10 minutes at 94.5°C, 30 seconds at 97°C, and 1 minute at 59.7°C for 40 cycles. The threshold cycle (Ct) was automatically provided by the SDS2.2 software package (Applied Biosystems). Delta Ct values for each gene were calculated using the Ct for GAPDH and the formula delta Ct = Ct_{GAPDH}-Ct_{gene}.

Flow cytometry

In all experiments, a LSR II flow cytometer (BD Biosciences) and BD FACSDiva™ software version 6.0 were used to collect and analyze data. For cell surface antigen staining, cells were washed in FACS buffer (PBS, 0.1% FBS, 0.1% NaN_3), Fc-receptors were blocked by incubation in human FcR blocking agent for 15 minutes (Miltenyi Biotec), and antibodies were pre-mixed into a cocktail and then incubated with cells for 90 minutes at room temperature in the dark. Cells were then washed twice in FACS buffer, and DAPI was added (0.5 ng/ml final concentration) to each tube to allow for electronic exclusion of dead cells during analysis. A minimum of 100,000 events was acquired for each sample. Compensation was performed using antibody capture beads (anti-mouse kappa chain; BD Biosciences) incubated with the same antibodies as used in the experimental tubes, and the AutoComp software routine was employed. Ratios for mean fluorescence intensity (MFI) were calculated as follows: MFI of

viable cells stained with specific antibody / MFI of viable cells stained with an isotype-matched irrelevant antibody.

For intra-cellular staining, NK cells were treated with BD GolgiStop™ for 2 hours before cell staining to inhibit protein transport. Cells were then fixed in 4% formyl saline fixation buffer and stained with a BD anti-human perforin antibody in 0.1% BD Cytofix/Cytoperm™ solution according to manufacturer's instructions.

Cytometric Bead Array (CBA), Luminex®, and ELISA assays

Levels of soluble proteins were quantified in supernatants from cultured cells using CBA, Luminex®, and ELISA assays. In the CBA assay, analyte standards were multiplexed to contain a mixture of predetermined amounts of 23 analytes and were used to prepare serial dilutions ranging from 5 to 5000 pg/ml. Assays were performed according to the manufacturer's instructions (BD Biosciences), and data were collected on a LSR II flow cytometer using BD FACSDiva™ software. BD FCAP Array™ software was used to fit standard curves to the data obtained from the analyte standards and to calculate absolute concentrations for the 23 analytes from the standard curves. In the Luminex® assay, culture supernatants were examined for levels of soluble proteins using the Milliplex MAG 30-plex Human Cytokine/Chemokine Panel from Millipore according to the manufacturer's protocol. TGFβ1, IFNγ, and IL-6, when tested individually, were assayed in triplicate with ELISA kits following the kit instructions (eBioscience). Cytokine concentrations were determined by referring to a standard curve and expressed as pg/ml.

Western blotting

Treated cells were quickly lysed in lysis buffer (Invitrogen). An equal amount of protein (20 μg) was loaded in each well, separated by SDS-PAGE, and transferred to nitrocellulose membrane, followed by probing overnight with anti-human STAT3, phospho-STAT3, SMAD2/3, phospho-SMAD2/SMAD3, SMAD1/5/8, phospho-Smad1/5/8, and β-actin. Blots then were processed with the appropriate secondary antibody (1:2000) and visualized.

Supplemental Figures

Supplemental Figure 1. Neuroblastoma cell line CHLA-255-Fluc conditioned medium (CM) stimulates monocytes to secrete IL-6 and IL-10 but not IL-2, IL-15, IL-12p70, or IFNγ. Purified monocytes ($5x10^5$ cells/ml) were cultured for 24 hours with CM generated after 72 hours by CHLA-255-Fluc neuroblastoma cells ($5x10^5$ cells/ml), and then cytokines in monocyte supernatants were quantified with the Milliplex MAG 30-plex (Luminex®) Human Cytokine/Chemokine Panel (mean $\pm$ SD for 3 replicate cultures). This experiment is representative of 3 experiments.

Supplemental Figure 2. Lenalidomide enhances ADCC by NK cells against neuroblastoma cell lines. (A and B) NK cells isolated from PBMC were cultured ($1x10^4$ cells/0.1 ml/well) for 72 hours with IL-2 alone (10 ng/ml) or with added lenalidomide as indicated, and then direct cytotoxicity and ADCC (E:T ratio = 2:1) with ch14.18 (0.1 µg/ml) were quantified after 6 hours of co-culture with CHLA-255-Fluc or LA-N-1 neuroblastoma cells using the calcein-AM/DIMSCAN assay (mean $\pm$ SD for 8 replicate cultures for each condition). T-test P-values *, $P<0.05$; **, $P<0.01$.

Supplemental Figure 3. Suppression of IL-2 activation of NK cells by neuroblastoma /monocyte CM (CM) is removed by depletion of IL-6 and TGFβ1. (A) Direct cytotoxicity and (B) ADCC mediated by NK cells against CHLA-255-Fluc cells. CM that contained IL-6 (385 pg/ml) and TGFβ1 (525 pg/ml) was generated by co-culture of monocytes and CHLA255-Fluc neuroblastoma cells in transwells for 3 days and then was depleted of IL-6 (CM-IL-6), TGFβ1 (CM-TGFβ1), or both with anti-IL-6 and anti-TGFβ1 mAbs (0.2ug/ml, Novus Biologicals) and protein A/G PLUS-agarose (Santa Cruz Biotech, 20ul per reaction). These CM were then tested for suppression of activation of purified NK cells by IL-2 or IL-2 with lenalidomide as described in Figure 2 (C and D). After 72 hours of culture, NK cell cytotoxicity and ADCC were

measured using a luciferase assay. NK cells were cultured with 5,000 CHLA255-Fluc target cells at an E:T ratio of 1:1 for 6 hours in 96-well white plates (Promega). Luciferin (2.5 µl of 5mg/ml stock) was added, allowed to react for 10 minutes, and then luminescence was quantified with a GloMax®-Multi Detection System (Promega). P-value *, P<0.05.

Supplemental Figure 4. TGFβ1 suppresses expression of NK cell NKG2D, NKp46, CD16, CD56, and CD107a. Purified NK cells were cultured with IL-2 (10 ng/ml) and TGFβ1 (10 ng/ml) without and with lenalidomide at indicated concentrations for 72 hours, and then expression of cell surface molecules was analyzed by flow cytometry. Confirmatory results were obtained from 1 additional experiment. T-test P-values *, P<0.05; **, P<0.01.

Supplemental Figure 5. Lenalidomide inhibits TGFβ1 induced SMAD2/3 translocation in NK cells. Purified NK cells were pre-incubated with lenalidomide (1 and 10 µM, 20 minutes), treated with TGFβ1 (10 ng/ml) for 20 minutes, and then analyzed for phosphorylated SMAD2/3 in nuclei. (A) Fluorescence microscopy. NK cells were fixed in 1% paraformaldehyde and permeabilized with 0.1% BD Cytofix/Cytoperm™ solution according to manufacturer's instructions. Cells were then incubated with anti-human pSMAD2/3 antibody (1:1000) followed by Alexa-fluor 488 goat anti-mouse IgG1 secondary antibody (1:1000) each for 1 hour at 4°C (Cell Signaling). The images were captured by the IPLab4.0 wide-field microscope (Zeiss). Original magnification is x250. (B) Quantification of nuclear staining with Cellomics imaging analysis (mean ± SD for 3 replicate cultures for each condition). This experiment is representative of 3 experiments. T-test P-values, no lenalidomide vs. lenalidomide *, P<0.05; **, P<0.01.

Supplemental Figure 6. Lenalidomide inhibits SMAD2/3 and STAT3 luciferase reporters in neuroblastoma cells in NOD/SCID mice. (A) CHLA-255 cells (10^6) transfected with Renilla

luciferase (hRluc) for tumor cell quantification and a SMAD2/3 firefly luciferase (Fluc) reporter for pathway activity were injected subcutaneously into the shoulder areas of NOD/SCID mice in 25% (v/v) BD Matrigel® Matrix Growth Factor Reduced. Treatment with lenalidomide (50 mg/kg/day intraperitoneal, daily x7 days and then 5 days/week) started after imaging and randomization on Day 3. Bioluminescence images were obtained on day 13 for SMAD2/3 Fluc and day 14 for hRluc. Quantification of the Fluc reporter signal is expressed as the ratio of the Fluc reporter signal to the hRluc signal. (B) CHLA-255 cells (10^6) transfected with hRluc for tumor cell quantification and a STAT3 Fluc reporter for pathway activity were injected subcutaneously into the shoulder areas of NOD/SCID mice 25% (v/v) BD Matrigel® Matrix Growth Factor Reduced. Treatment with lenalidomide (50 mg/kg/day intraperitoneal, daily x7 days and then 5 days/week) started after imaging and randomization on Day 3. Bioluminescence images were obtained on day 15 for STAT3 Fluc and day 16 for hRluc. Quantification of the Fluc reporter signal is expressed as the ratio of the Fluc reporter signal to the hRluc signal (mean $\pm$ SD for 2 tumors per mouse totaling 10 tumors for each condition). T-test P-values, no lenalidomide vs. lenalidomide *, $P<0.05$.

Supplemental Figure 7. Removal of NK cells from PBMC co-injected with tumor cells decreases the anti-neuroblastoma activity in NOD/SCID mice of lenalidomide alone, ch14.18 alone, and lenalidomide combined with ch14.18. As part of the experiment shown in Figure 6A, CHLA-255-Fluc neuroblastoma cells (10^6) and PBMC (0.25×10^6) depleted of NK cells (PBMC-NK) were co-injected into NOD/SCID mice near the right and left shoulders in 25% (v/v) BD Matrigel® Matrix Growth Factor Reduced on day 0. Treatment began on day 1 (after imaging and randomization) with lenalidomide alone (50 mg/kg/d intraperitoneal days 1-9 and 12-16), anti-GD2 ch14.18 alone (15 µg/mouse intravenous days 3, 5, 8, and 12), or the combination of lenalidomide and ch14.18 using the same doses and schedules. There were five mice in each treatment group. (A) Growth of neuroblastoma tumors based upon

bioluminescence imaging of tumors of each mouse. Bioluminescence imaging measurements were performed on the left and right sides of each mouse and were collected 1, 8, and 15 days after injection of tumor cells for calculation of area under the curve (AUC) for each group. Mice that were treated with the combination of lenalidomide and ch14.18 had smaller AUC when PBMC versus PBMC-NK were co-injected (P=0.05). (B) Survival of mice in different groups. Survival time, which is defined as the days from tumor cell injection until the mouse is sacrificed due to tumor size or the end of the experiment (day 56), was greater for mice that received PBMC vs. PBMC-NK and treated with the combination of lenalidomide and ch14.18 (P = 0.006).

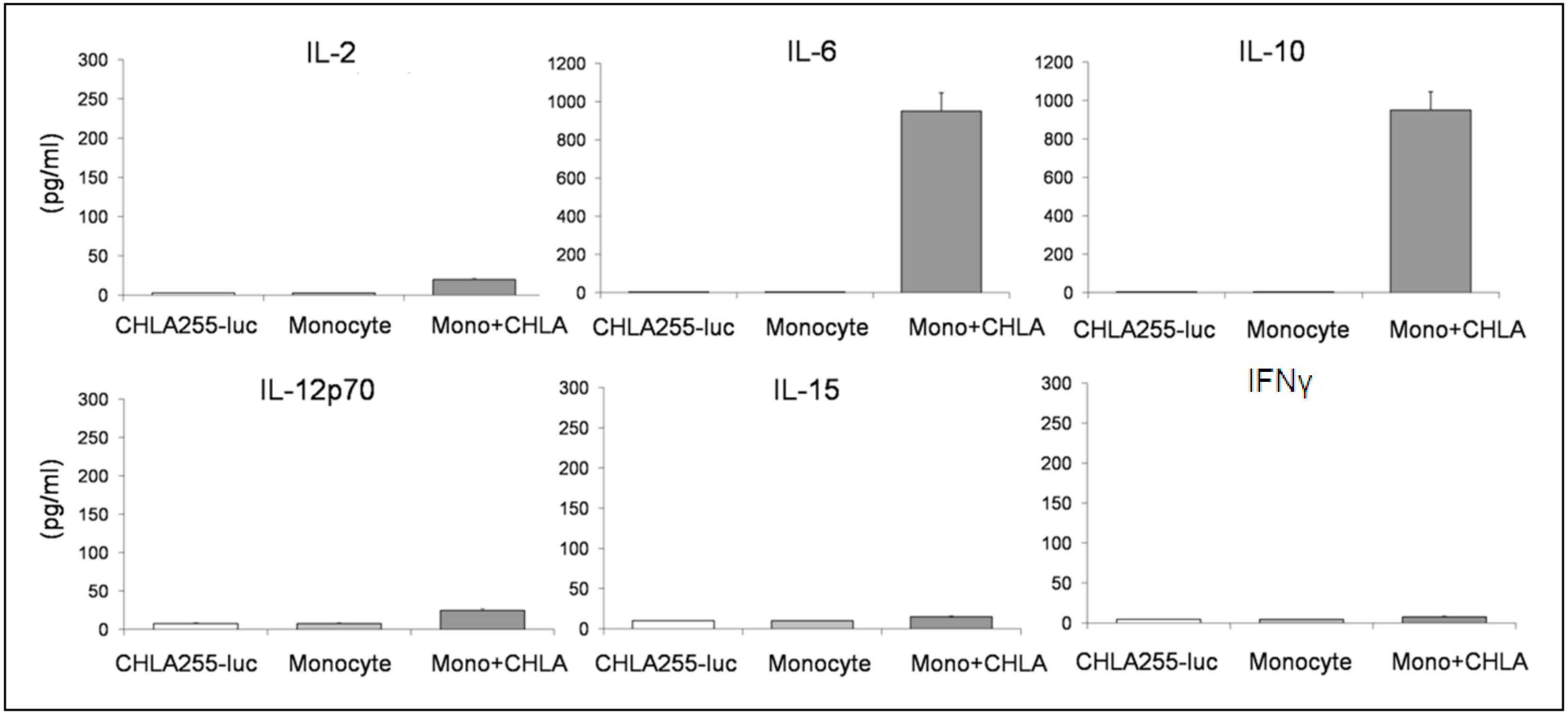

Supplemental Figure 1

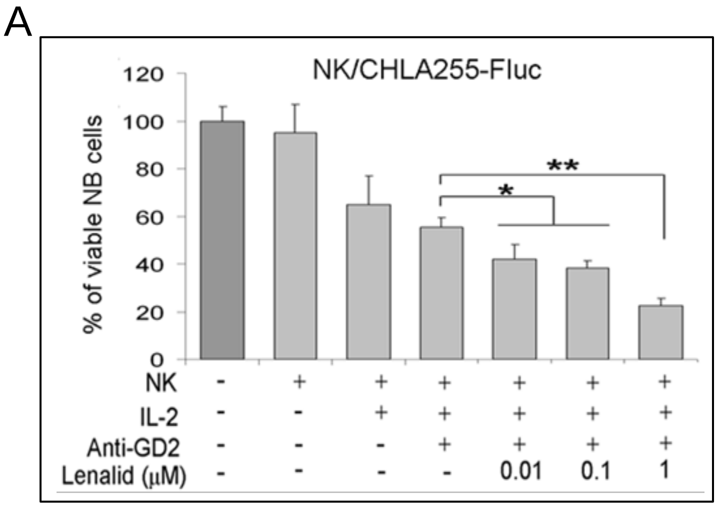

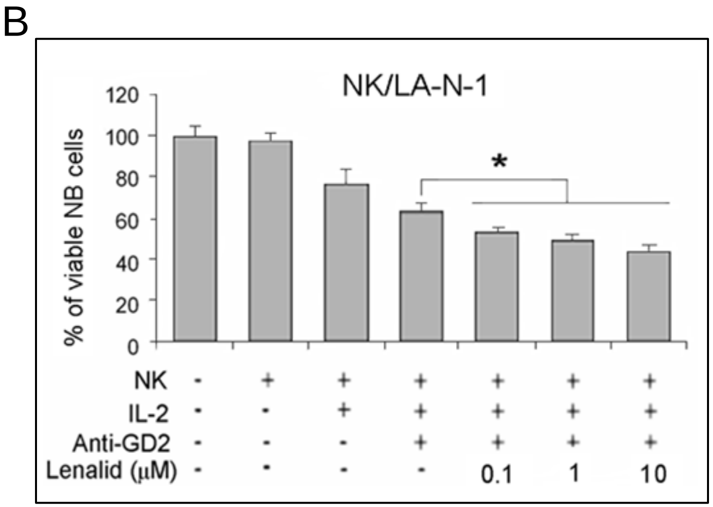

Supplemental Figure 2

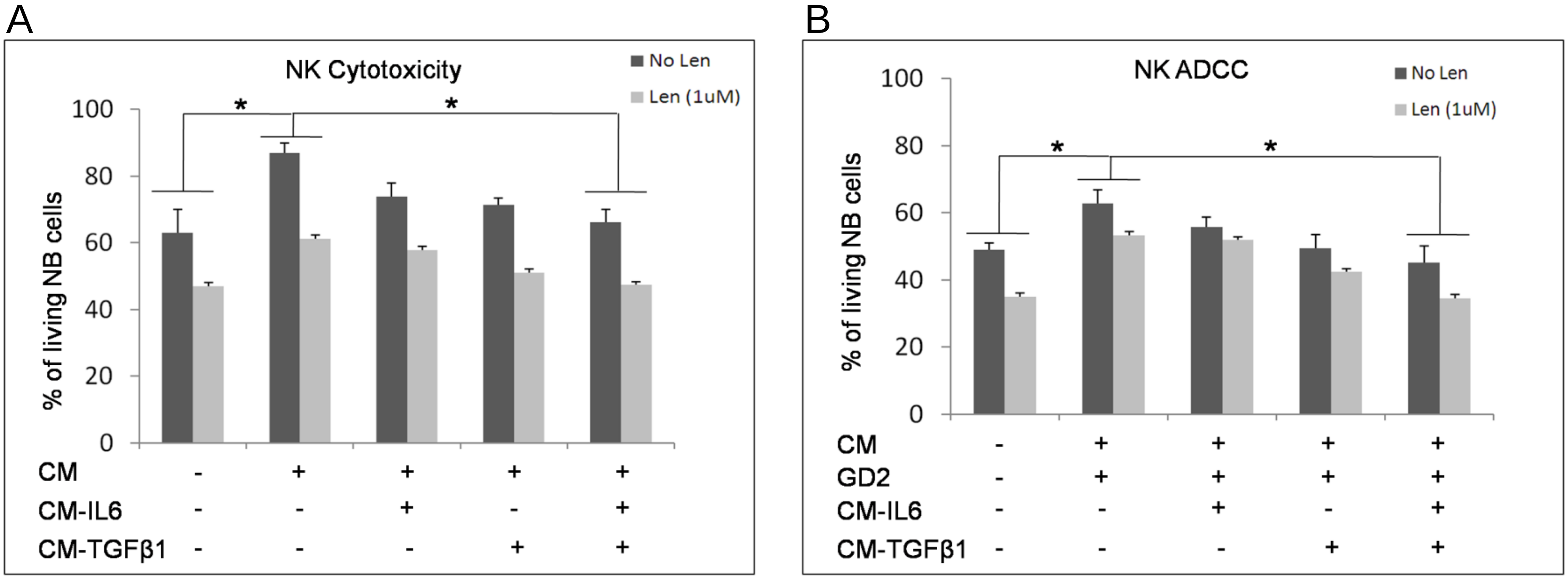

Supplemental Figure 3

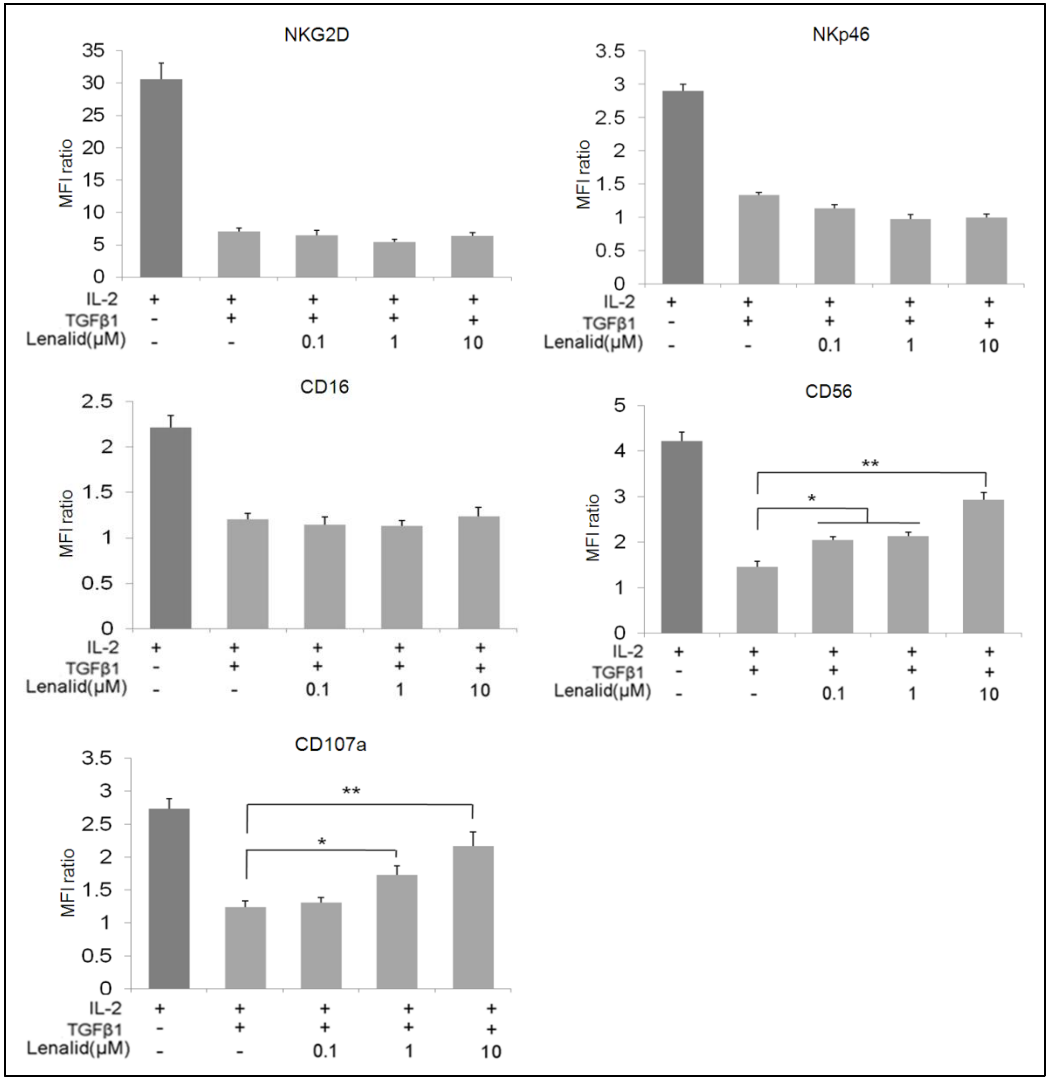

Supplemental Figure 4

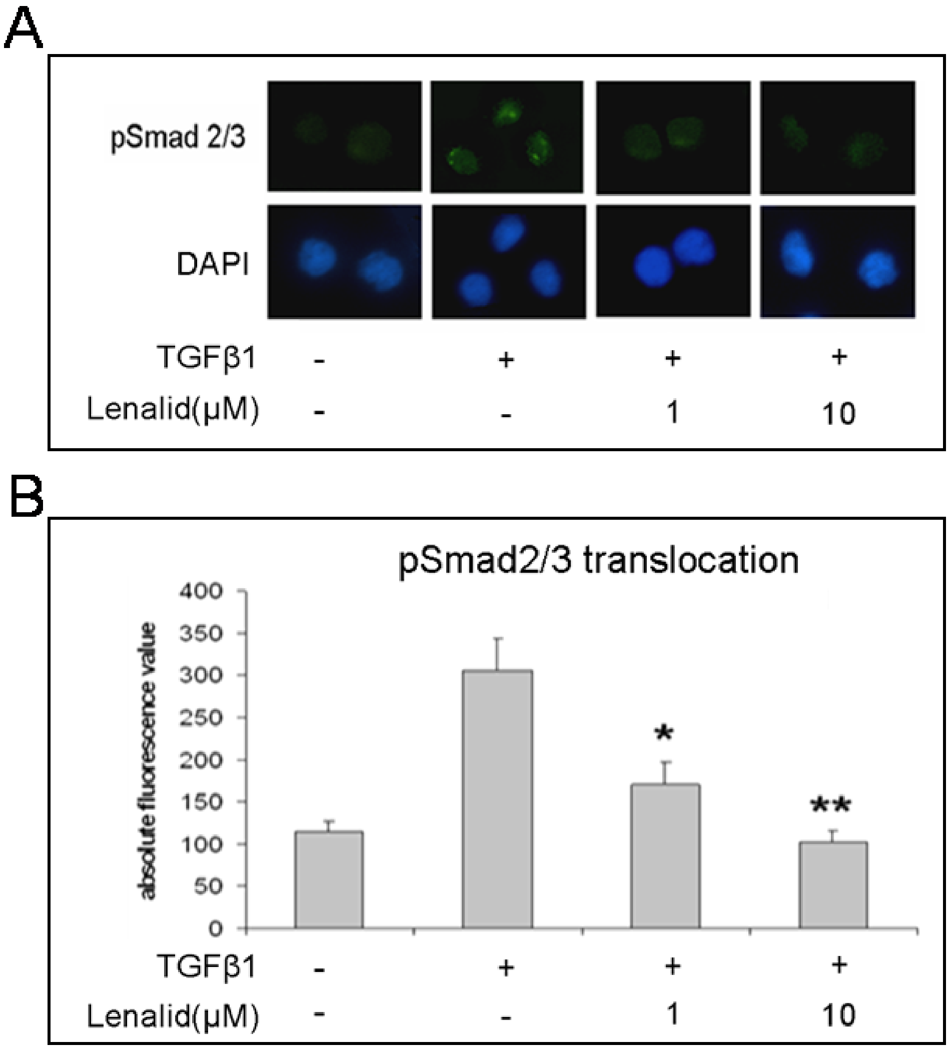

Supplemental Figure 5

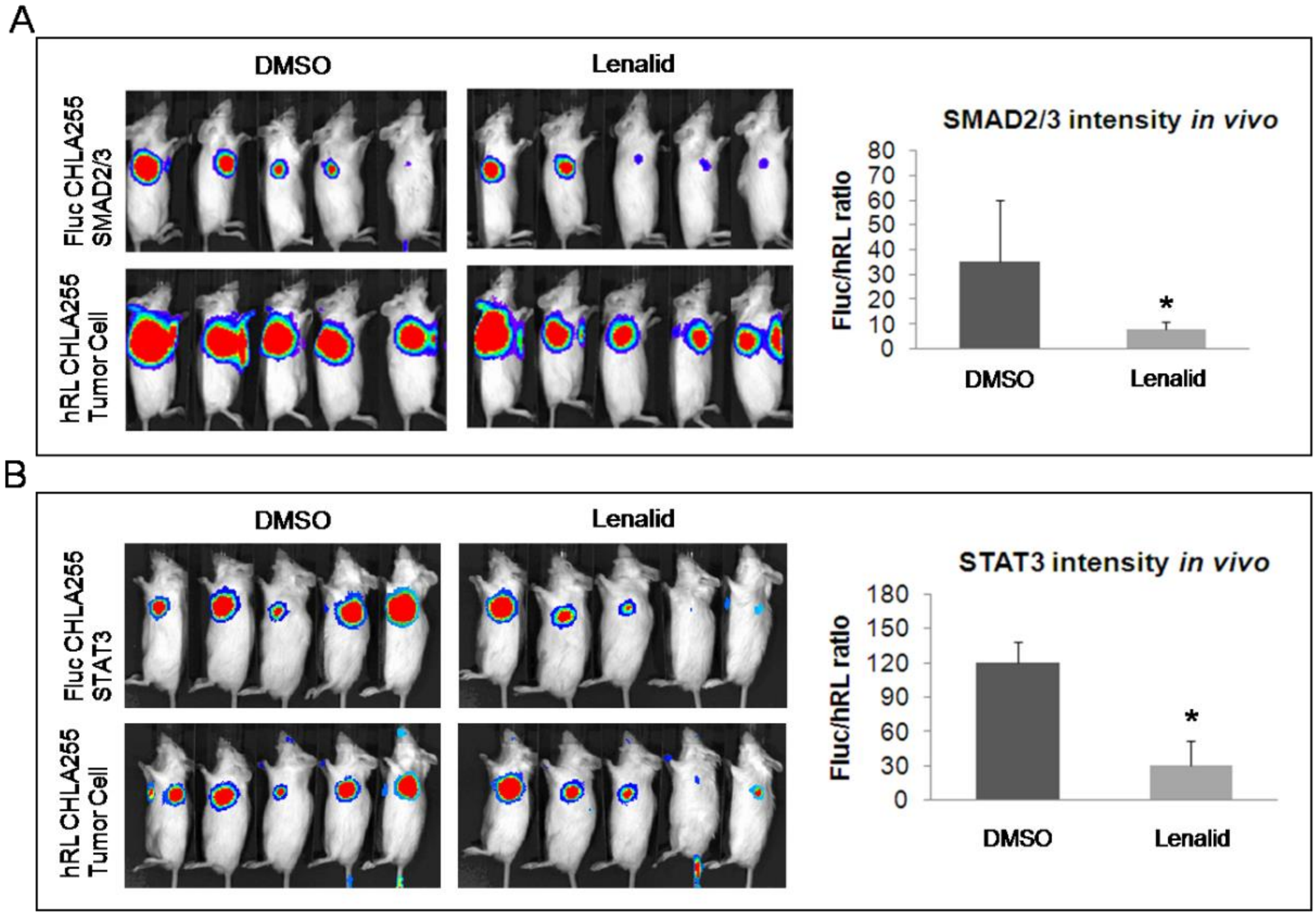

Supplemental Figure 6

A

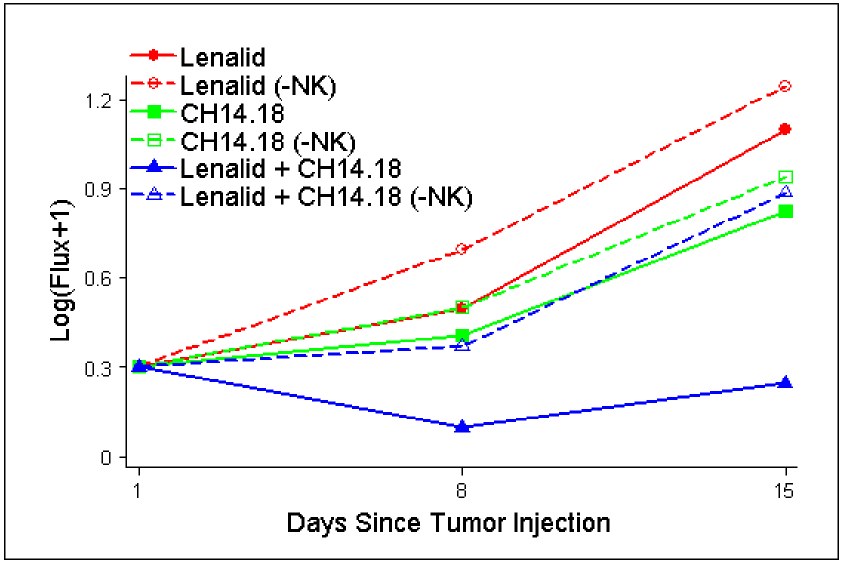

B

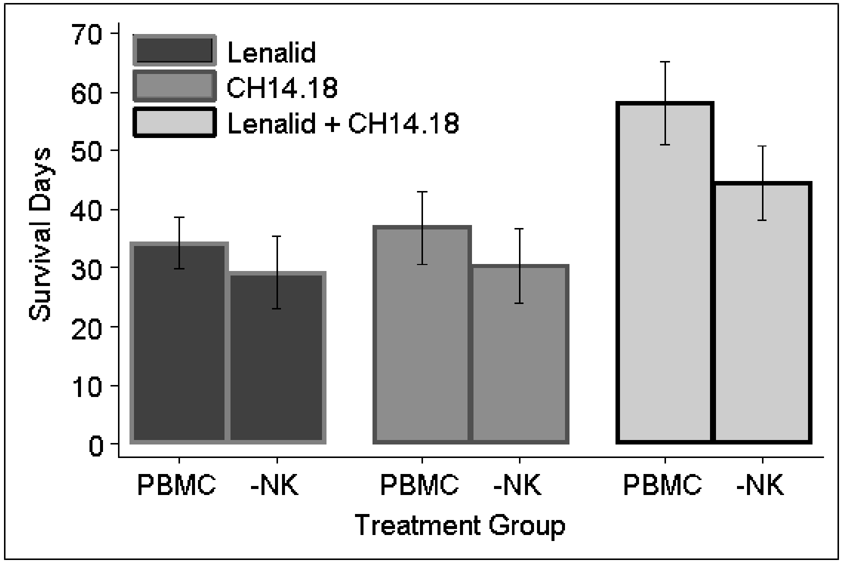

Supplemental Figure 7
